# Application of Bipolar Membrane Electrodialysis in Environmental Protection and Resource Recovery: A Review

**DOI:** 10.3390/membranes12090829

**Published:** 2022-08-24

**Authors:** Yu Luo, Yaoxing Liu, Jiangnan Shen, Bart Van der Bruggen

**Affiliations:** 1 College of Environmental and Resource Sciences, College of Carbon Neutral Modern Industry, Fujian Key Laboratory of Pollution Control & Resource Reuse, Fujian Normal University, Fuzhou 350007, China; 2 Department of Chemical Engineering, ProcESS-Process Engineering for Sustainable System, KU Leuven, Celestijnenlaan 200F, B-3001 Leuven, Belgium; 3 College of Chemical Engineering, Zhejiang University of Technology, Hangzhou 310014, China; 4 Faculty of Engineering and the Built Environment, Tshwane University of Technology, Private Bag X680, Pretoria 0001, South Africa

**Keywords:** bipolar membrane electrodialysis, acid and base, carbon dioxide, ammonia nitrogen, wastewater

## Abstract

Bipolar membrane electrodialysis (BMED) is a new membrane separation technology composed of electrodialysis (ED) through a bipolar membrane (BPM). Under the action of an electric field, H_2_O can be dissociated to H^+^ and OH^−^, and the anions and cations in the solution can be recovered as acids and bases, respectively, without adding chemical reagents, which reduces the application cost and carbon footprint, and leads to simple operation and high efficiency. Its application is becoming more widespread and promising, and it has become a research hotspot. This review mainly introduces the application of BMED to recovering salts in the form of acids and bases, CO_2_ capture, ammonia nitrogen recovery, and ion removal and recovery from wastewater. Finally, BMED is summarized, and future prospects are discussed.

## 1. Introduction

Electrodialysis (ED) is a membrane separation technology, and the ion exchange membrane present in ED is divided into anion-exchange membrane (AEM) and cation-exchange membrane (CEM). CEM and AEM are selective for cation and anion transfer [1,2,3] under an electric field [4,5,6]. ED is widely used in (bio)chemistry, food processing, wastewater treatment, chemical recycling, and the removal of toxic components, but the high energy consumption limits its wider development [7].

In 1956, Frilette [8] made a breakthrough in membrane separation technology. He was the first to propose a bipolar membrane (BPM) [9], which is a special ion exchange membrane that consists of an AEM and a CEM [10]. Their combined thickness is less than 10 nm [11,12]. Under the reverse bias of a DC electric field, H^+^ and OH^−^ can be generated by dissociating H_2_O [13]. BPM is used in the (bio)chemical industry, as well as for food processing, environmental protection, and energy conversion and storage, because it can acidify organic salts in situ without causing salt contamination, and converts the cations into a base (which can be reused to produce organic salts) [14].

**Figure 1:**
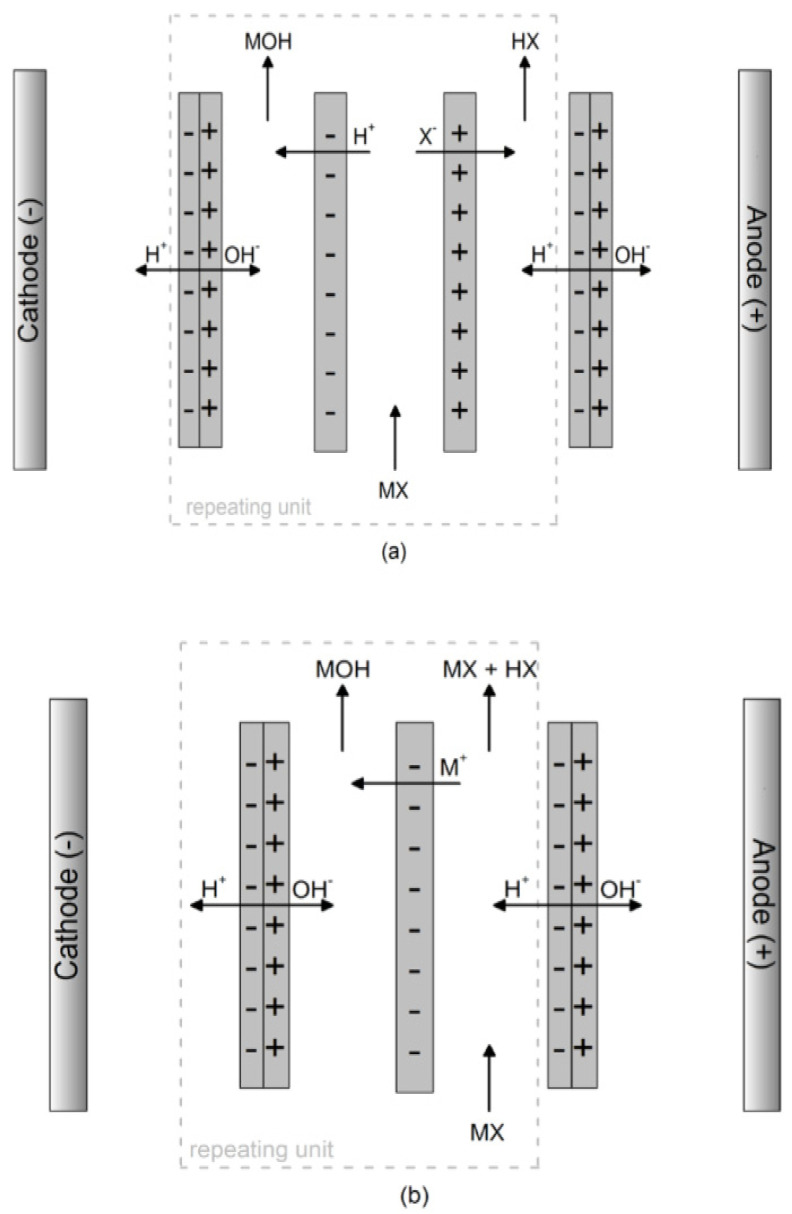

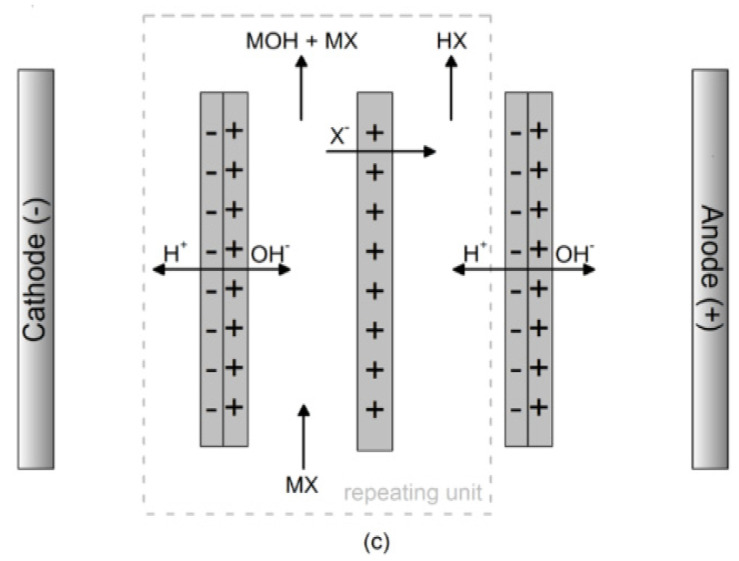
Schematic diagram of BMED: (**a**) three-chamber BMED; (**b**) two-chamber: base-BMED; and (**c**) two-chamber: acid-BMED [17].

Over the past 20 years, bipolar membrane electrodialysis (BMED), a system combining ED and BPM, has been developed for its ability to convert anions and cations into acids and bases, respectively. BMED is composed of BPM, CEM, and AEM in a certain arrangement [15] and has three basic membrane structures (three-chamber BMED; two-chamber BMED containing acids or base) depending on the application. Among them, the three-chamber BMED (Figure 1a) contains CEM, AEM, and BPM in each membrane repeating unit, and the benefit of it is that the salts can be separated and recovered as acids and bases [13]. Two-chamber BMED includes base-BMED (Figure 1b) and acid-BMED (Figure 1c); the difference from the three-chamber BMED is that an AEM or CEM is removed from each membrane repeating unit, resulting in only base or acid being produced. Base-BMED (Figure 1b) can be used to treat weak acid salts (organic acid salts) for obtaining a relatively pure base; acid-BMED (Figure 1c) can be used to convert weak base salts into a relatively pure acid. Moreover, two-chamber BMED has advantages, such as requiring a smaller membrane area (per repeating unit), simple operation, high efficiency, low energy consumption, and only with two outlet streams [13]. However, the base generated by the cathode in the two-chamber BMED is easily transported to an acid chamber through the AEM, neutralizing the acid, therefore reducing the amount of acid and current efficiency [16].

In a three-chamber BMED, the basic units are repeatedly placed between the electrodes. The salt solution flows in the chamber between the AEM and the CEM; the CEM is not directly in contact with the acid, and the AEM is not directly in contact with the base, so the durability of the membrane is significantly increased compared to in the two-chamber BMED [18]. This also overcomes the disadvantage of ion leakage that was present in the two-chamber BMED. When a direct current is applied, water will dissociate into equal amounts of H^+^ and OH^−^ in the BPM; the generated H^+^ forms HX with the X^−^ ions provided by the salt solution, OH^−^ and M^+^ ions provided by the salt solution form MOH, and the corresponding acids and base are obtained. It was shown that concentrations of acids and base as high as 6 M can be produced in a three-chamber BMED. However, its energy consumption is relatively high due to the resistance of the salt solution increasing with the ion migration, which resulted in a high voltage under the condition of a stable current density.

Compared with other electrochemical technologies, BMED has the characteristics of lower energy consumption and higher economic efficiency. The water dissociation potential (0.828 V) required in BPM is much lower than that of water electrolysis on electrodes (2.057 V). The electrolysis reaction is compared as follows (assuming that 1 mol of acid and base are both generated) [19]:

The overall electrolysis reaction on an electrode:

(1) $${3H}_{2}O\to {1/2O}_{2}+H_{2}+{2H}^{+}+{2\mathrm{OH}}^{-},\;\mathrm{theoretical}\;\mathrm{potential}\;2.057\;V$$

cathodic reaction:

(2) $${2H}_{2}O+2e\to H_{2}+{2\mathrm{OH}}^{-},\;\mathrm{theoretical}\;\mathrm{potential}\;0.828\;V$$

anodic reaction:

(3) $$H_{2}O\to {1/2O}_{2}+{2H}^{+}+2e,\;\mathrm{theoretical}\;\mathrm{potential}\;1.229\;V$$

Bipolar membrane water dissociation reaction:

(4) $$2H_{2}O\begin{array}{c} \overset{k_{1}}{\to }\\ \underset{k_{-1}}{\leftarrow } \end{array}H_{3}O^{+}+2{\mathrm{OH}}^{-},\;\mathrm{theoretical}\;\mathrm{potential}\;0.828\;V$$

where k_1_ is the water dissociation rate constant, and k_−1_ is the water recombination rate constant.

The water dissociation reaction in BPM is described as a two-stage protonation–deprotonation reaction [20]. The protonation–deprotonation mechanism suggests the possibility of generating H^+^ and OH^−^ ions via proton transfer reactions between water and immobilized charged groups [21,22,23], involving the participation of catalysts in the dissociation of water, and it is believed that the water dissociation reaction depends on the catalytic activity of the immobilized group. Therefore, the reaction in Equation (4) can be divided into the following processes:

(5) $$B+H_{2}\;O\begin{array}{c} \overset{k_{1}}{\to }\\ \underset{k_{-1}}{\leftarrow } \end{array}{\mathrm{BH}}^{+}+{\mathrm{OH}}^{-}$$

(6) $${\mathrm{BH}}^{+}+H_{2\;}O\begin{array}{c} \overset{k_{2}}{\to }\\ \underset{k_{-2}}{\leftarrow } \end{array}B+H_{3}O^{+},$$

where B is a weak base, BH^+^ is the catalytic center (i.e., fixed charged groups of AEM), k_1;2_ is the forward rate constant, and k_−1;−2_ the backward rate constant.

(7) $$\mathrm{AH}+H_{2}O\begin{array}{c} \overset{k_{1}}{\to }\\ \underset{k_{-1}}{\leftarrow } \end{array}A^{-}+H_{3}O^{-}$$

(8) $$A^{-}+H_{2}O\begin{array}{c} \overset{k_{2}}{\to }\\ \underset{k_{-2}}{\leftarrow } \end{array}\mathrm{AH}+{\mathrm{OH}}^{-},$$

where A^−^ is the catalytic center in the membrane.

If the dissociated ions combine with the ions in solution, the overall reaction of BPM can also be written as:

(9) $$\mathrm{MX}+H_{2}O\to \mathrm{MOH}+\mathrm{HX},$$

where MX refers to a generic electrolyte and MOH and HX to its corresponding base and acid, respectively.

The energy consumption of electrohydrolysis for 1 mol of water is 198.5 KJ/mol on electrodes, and 79.9 KJ/mol in BPM. H_2_ and O_2_ are not generated during the BMED process, which has no effect on the electrode. This gives BMED better application prospects compared to other electrochemical technologies.

This review mainly introduces the application of BMED in terms of recovering salts in the form of acids and bases, CO_2_ capture, ammonia nitrogen production and recovery, and ion removal and recovery from wastewater.

## 2. Recovering Salts in the Form of Acids and Bases

### 2.1. Inorganic Acid and Base Recovery from Salt Solutions

**Figure 2:**
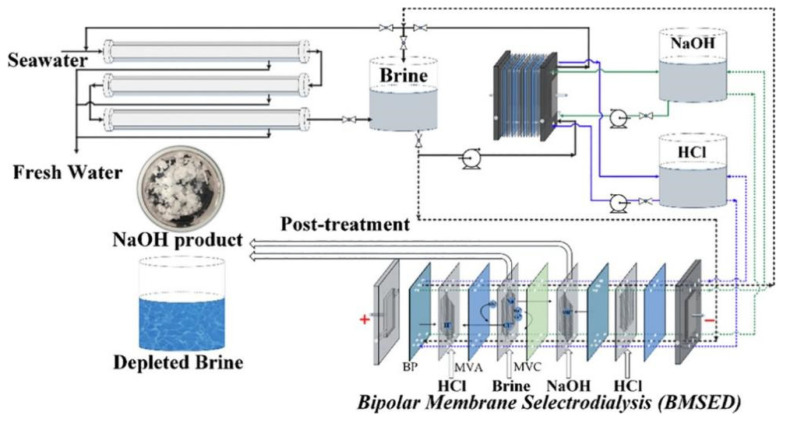
Schematic diagram of the BMSED process [30].

**Figure 3:**
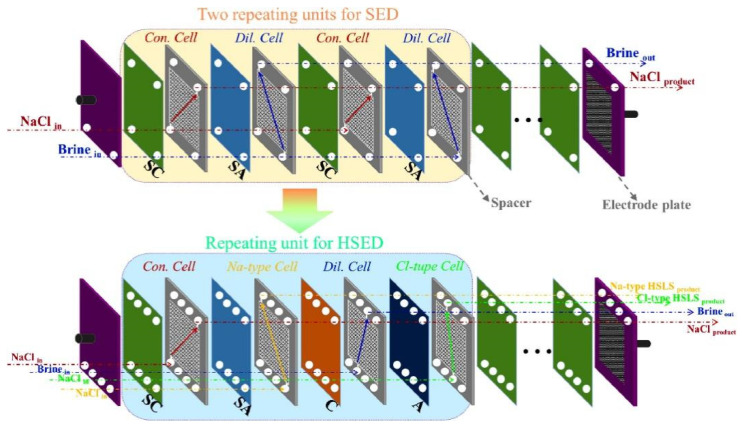
Structural diagram of SED and HSED stacks [31].

BMED technology is often used to treat high-salt wastewater. A large number of previous studies reported acid and base recovery from NaCl and Na_2_SO_4_ containing wastewater. For example, Gao et al. [24] used a BMED system to recover acid and base from NaCl wastewater generated from the process of generating HZSM-5 zeolite. Yang et al. [18] used a BMED to treat seawater reverse osmosis (RO) concentrate; 1 mol/L of mixed acid and base was continuously produced at a constant current density of 57 mA/cm^2^, and the produced acid was used for pH adjustment of RO process and controlling of RO membrane fouling. In addition, the combination of BMED with other technologies is also used to treat waste acid, waste base, and other solutions. Zhuang et al. [25] recovered acid and aluminum in the form of Al(OH)_3_ from a waste acid generated from the aluminum foil industry using a combined BMED with diffusion dialysis. When a RO-ED-BMED combination process was used to treat brine (Na_2_SO_4_), NaOH and H_2_SO_4_ were recovered and pure water was generated [26]. Reig et al. [27] integrated an ED + BMED system to treat high-salt wastewater. ED was used to concentrate salts and BMED was used to convert salts to acids and bases, and 1.9 mol/L of NaOH and 2.0 mol/L of HCl were finally recovered with consumption of 1.7 kW·h/kg [28]. Badruzzaman et al. [29] proposed a new integrated membrane system(IMS)–BMED technology for treating RO high-salt wastewater, and it was found that the IMS–BMED system had certain advantages in terms of investment costs and operating costs. Chen et al. [30] first proposed a new process called BPM selective eletrodialysis (BMSED) to treat concentrated saline, which works by combining selective electrodialysis (SED) and BMED processes (Figure 2). The results show that the selective permeability of Na^+^/Ca^2+^ and Cl^−^/SO_4_^2−^ are 5 to 10 and 50 to 60, respectively; the concentrations of NaOH and HCl increased to 2.2 and 1.9 mol/L, respectively, and the purity of NaOH and HCl were both close to 99.99%. Chen et al. [31] proposed a novel innovative hybrid selective ED (HSED) and a selective BMED (SBMED) (Figure 3) to treat seawater brine. The HSED process was used to enrich major divalent cations and anions and the SBMED process was used to produce acids and bases without any purification pretreatment. When the combination technology of ozonation and BMED was used to treat textile wastewater, 90% of color, 37% of chemical oxygen demand, and more than 90% of salt could be removed [32].

**Figure 4:**
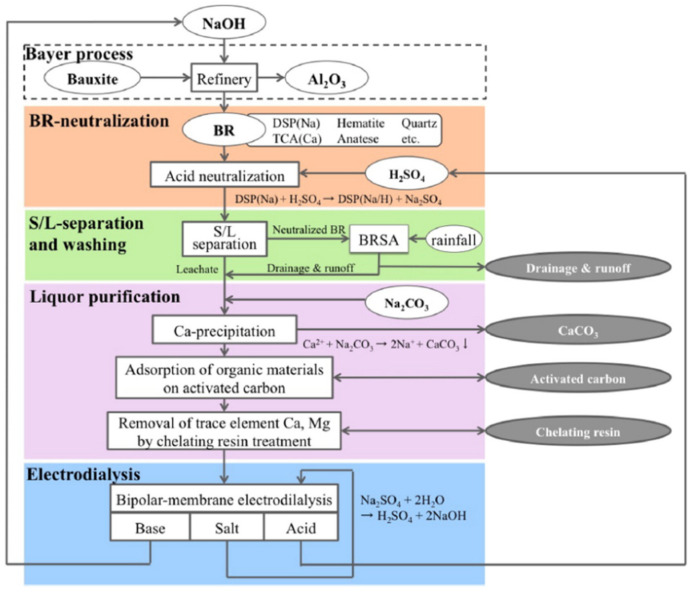
Process of in situ remediation of bauxite residue by sulfuric acid neutralization [34].

Gonzalez et al. [33] determined and analyzed the scope and feasibility of BMED for recovering high-purity LiOH from an LiCl solution. The results showed that when the initial LiCl concentrations were 0.5 wt. % and 14 wt. %, the concentrations of obtained LiOH solutions were 3.34 wt. % (purity 96.0%) and 4.35 wt. % (purity 95.4%), respectively. However, when the concentration of LiCl was in the range of 25–34 wt. %, Cl^−^ is easily diffused into the LiOH solution; also, the phenomenon of OH^−^ leakage occurred with LiOH concentration increase. Currently, this can be addressed by asymmetric BPM, or by avoiding high HCl concentrations and current density. In a previous study by Kishida et al. [34], BMED was used to generate NaOH and H_2_SO_4_. The NaOH was used in the Bayer refinery and H_2_SO_4_ was used to neutralize bauxite residues. In their study, the purpose of treating waste with waste was realized and the main treatment process was as shown in Figure 4.

**Figure 5:**
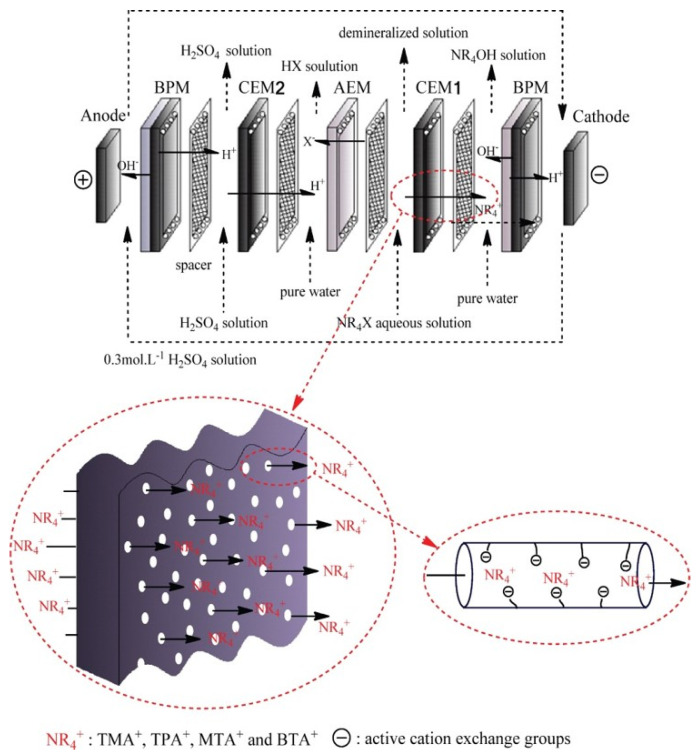
Configuration of the elementary cell for the preparation of NR_4_OH using a four-chamber bipolar membrane electrodialysis system. BPM, bipolar membrane; AEM, anion-exchange membrane; CEM (1,2), cation-exchange membrane; NR_4_X, quaternary ammonium halide (TMACl, TPABr, MTACl, and BTACl) [39].

The study by Erkmen et al. [35] proved that BMED is an environmentally friendly and easy-to-implement technology for the production of HF and NaOH from a NaF solution: the purity of HF and NaOH were 99.7% and 99.1%, respectively, with a current efficiency of 98.5%. Compared with the reaction of acid-grade fluorspar (CaF_2_) with H_2_SO_4_ and NaOH to produce HF in the chlor-base process, BMED has more commercial value because the traditional production method of choline hydroxide has disadvantages such as low product purity, low yield, and high energy consumption. In the study by Yao et al. [36], 86.8% of choline chloride was converted to choline hydroxide in a BMED system with low energy consumption (0.74 kWh kg^−1^) and high current efficiency (84.6%) [37,38]. Shen et al. [39] produced high-purity (Cl^−^/Br^−^ content < 500 ppm) quaternary ammonium hydroxides (Figure 5) in a novel four-chamber BMED; the details of the BMED are shown in Figure 4. Compared with traditional quaternary ammonium hydroxide synthesis methods, such as quaternary ammonium chloride reaction, ion exchange, and electrodialysis, there is no secondary pollution created in the BMED process.

**Figure 6:**
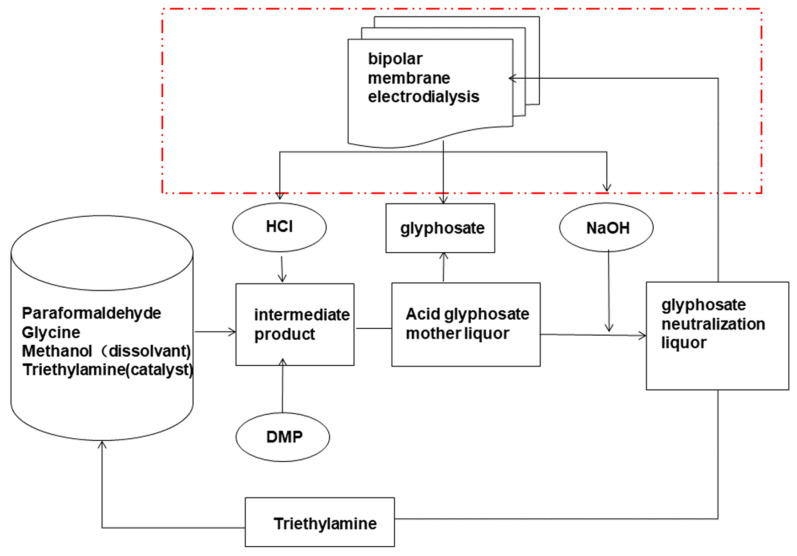
A simple technological flow diagram of the glycine–dimethylphosphit process coupled with a BMED process [43].

In addition, BMED was used to treat some pollutants that are difficult to treat with traditional methods. Glyphosate (N-phosphonomethyl glycine) is a postemergent nonselective broad-spectrum herbicide used worldwide [40]. The glycine–dimethylphosphite and iminodiacetic acid processes are the two main methods for synthesizing glyphosate [41]. However, both methods produce large amounts of glyphosate and high-salinity liquid [42], the discharge of which without treatment will not only cause environmental pollution, but also a waste of resources [43]. In 2012, BMED was first used to treat a high-salt glyphosate solution [43] and the obtained acid-based solution could be used for producing glyphosate (Figure 6); however, the process remained at the laboratory stage, and the prepared acid–base concentrations were all less than 1 mol/L. A three-chamber BMED was also used by Shen et al. [44] to treat glyphosate wastewater, and glyphosate and high-concentration base/acid were recovered; the current efficiency of producing NaOH with a concentration of 2.0 mol L^−1^ was above 67% and the corresponding energy consumption was 2.97 kWh kg^−1^, when the current density was 60 mAcm^−2^. When the current density was 30 mAcm^−2^, the current efficiency increased and the energy consumption decreased (87.13% and 2.37 kWh kg^−1^, respectively). Compared to normal methods for treating glyphosate wastewater, BMED avoided the high energy consumption and low recovery of the evaporation method, and the high-salt residue of nanofiltration [45]. BMED was also used to treat pesticide wastewater [46], in which 99% of Na_2_SO_4_ was removed and 97% of 2,6-Difluorobenzamide was recovered.

From the above, we see that BMED has wide applications in treating different salt-containing wastewaters; however, high-salt wastewater is complex and contains various inorganic ions, organic substances, and heavy metals. Among them, hardness ions and heavy metal ions can be deposited on the surface of membranes, blocking the membrane channels and reducing the effective area of the membrane; too much organic matter will cause BPM swelling or adsorption by membranes, resulting in a decrease in selectivity [47]. Therefore, BMED influent generally requires low-concentration hardness ions (such as calcium and magnesium below 0.1 mg/L) and turbidity (below 1 mg/L) to protect its membrane components. In the future application of BMED, how to pretreat wastewater and develop a fouling-resistant membrane are ongoing topics of research.

### 2.2. Organic Acid–Base Recovery from Salt Solution

The BMED process is also used for the production and recovery of organic acids. Normally, organic acids are produced by chemical synthesis or fermentation [48]. The products generated by chemical synthesis are usually highly pure and concentrated [49,50], but the energy consumption is high, an expensive catalyst is needed [51], and some toxic and environmentally harmful reagents are used [52,53]; these limit the development of synthetic methods. The organic acids generated by fermentation are of high purity [54], but impurities such as sugar and mineral salts [55] often exist in the fermentation broth. To obtain a high-purity product, the impurities need to be removed using separation and purification technologies including precipitation [56], acidification, extraction [57,58,59], crystallization, distillation, and adsorption [60]; this consumes a large amount of chemical reagents and generates a large amount of waste liquid and residues that pollute the environment. Both of these methods fail to meet the requirements of modern chemistry, characterized by “design for the environment” and “green chemistry” [61]. To avoid the above disadvantages, ED [62] and ion exchange [63] are also used to produce organic acids. ED uses the principle of the selective permeability of AEM and CEM under the action of an electric field to produce organic acids from organic acid salt solutions. Since the ED system cannot generate H^+^, a large amount of acid needs to be added during the ED process, which leads to high costs. The main principle of ion exchange is to exchange ions through an ion exchange resin and convert them into organic acids, but the disadvantage is that the ion exchange resin is bulky and needs to be regenerated, resulting in a large amount of acid and alkali being consumed. In recent years, BMED has often been used to produce organic acids such as citric acid, fumaric acid, vitamin C, alpha-ketoglutaric acid, salicylic acid, gluconic acid, succinic acid, amino acids, and itaconic acid (IA) from a fermentation broth owing to its characteristics of simple operation, no addition of chemical reagents, and environmental friendliness [64]. Organic acids and bases can be generated directly by BMED through combining the H^+^ and OH^−^ generated by BMP and anions and cations in the fermentation broth. Table 1 shows the properties of ED, ion exchange, and BMED for obtaining organic acid from a fermentation broth.

In the study by Xu et al. [65,66], a two-chamber BMED was used to produce citric acid from sodium citrate solution. The effect of battery configuration and salty concentration on the energy consumption and electroacidification parameters were investigated; finally, citric acid with a concentration of 30 g/L was obtained. Cauwenberg et al. [67] also successfully recovered citric acid (concentration: 8.2–20.7 wt. %) from a sodium citrate solution (initial concentration: 15–18 wt. %) using a two-chamber EDBM with current density 40–100 mA cm^−2^. Sun et al. [68] produced citric acid from a fermentation broth using a two-chamber BMED. The effect of current density, initial sodium citrate concentration, and the structure of the acid and base chambers on BMED performance were investigated, and acid recovery of 97.1% was achieved under the conditions of initial sodium citrate 3.3 wt. % and current density 40 mA cm^−1^. Yu et al. [69] applied EDBM to the acidification process of sodium L-ascorbate and sodium 2-keto-L-guluronate, replacing the original sulfation process and ion exchange method in the production of vitamin C. The conversion rate was higher than 98% with an average current efficiency of about 70% and an electricity consumption of 1 kWh/kg of vitamin C. Szczygiełda et al. [70] produced α-ketoglutarate from a simulated solution using a two-chamber EDBM stack. Under the optimal conditions, the obtained alpha-ketoglutaric acid concentration, current efficiency, and energy consumption were 4.83 g/L, 71.8%, and 3.72-kW h/kg, respectively. Jiang et al. [71] produced morpholine (Mp) from a sulfate Mp solution with an energy consumption of 4.27 kW h/kg.

**Figure 7:**
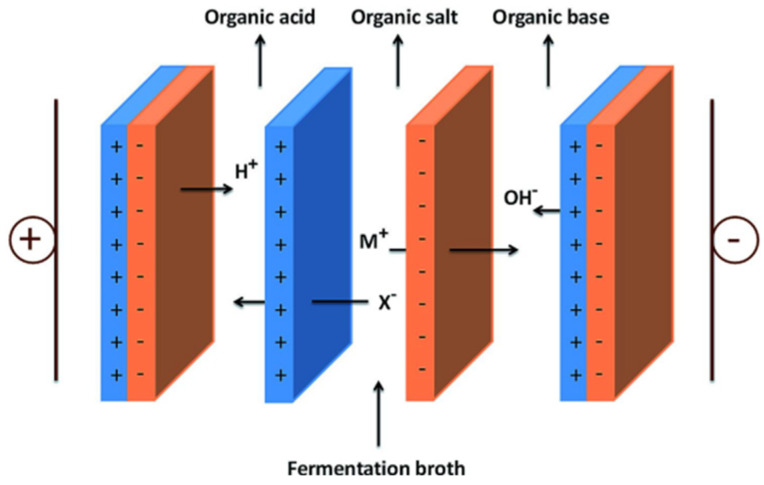
Schematic of the EDBM stack configuration for producing organic acids [72].

**Figure 8:**
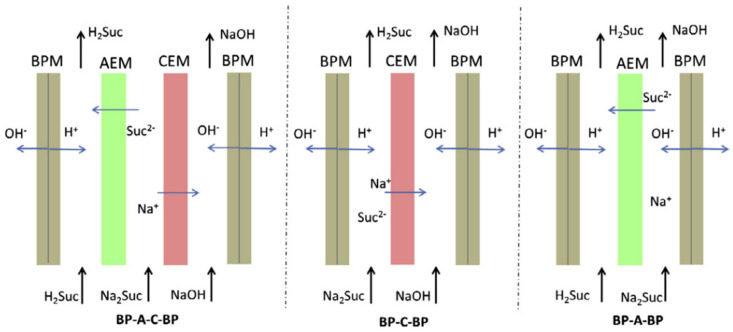
Schematic overview of the three configurations used to convert sodium succinate to succinic acid [73].

**Figure 9:**
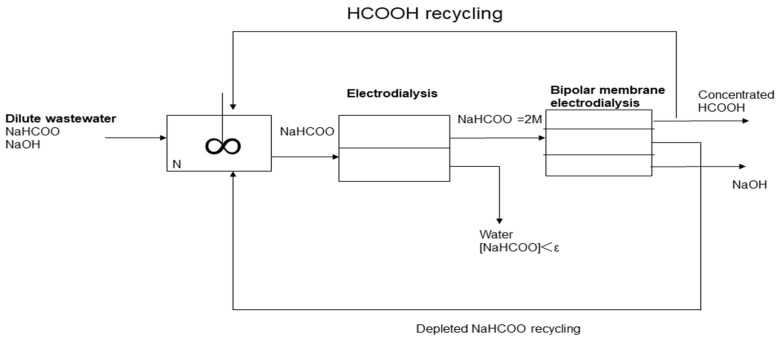
Global process for sodium formate splitting into formic acid and sodium hydroxide [74].

**Figure 10:**
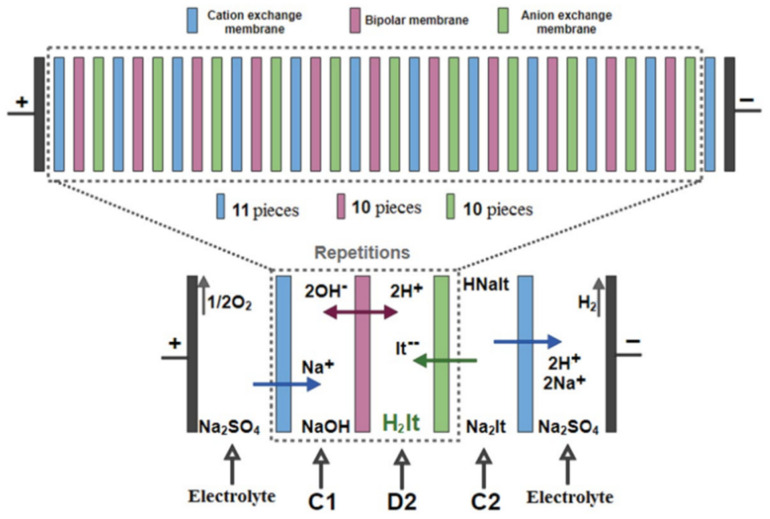
Scheme of the membrane module in the BMED and the predominant transport mechanisms during the separation, C_1_: NaOH compartment; D_2_: itaconic acid compartment; C_2_: itaconic acid sodium compartment [77].

Three-chamber BMED is also used to produce strong and weak organic acids from different solutions. A normal three-chamber BMED consists of a fermentation broth, base, and acid chamber, as shown in Figure 7 [72]. Fu et al. [73] employed different BMED to recover succinic acid from a sodium succinate solution. The properties of BMED with three different structures were investigated according to energy consumption, current efficiency, voltage drop across the stack, and yield concentration (Figure 8). It was found that the BMED could not work at a high current density and had a relatively high energy consumption. BPM-CEM-BPM BMED has a low energy consumption and high acid concentration, but it is difficult to obtain high-purity succinic acid. However, BPM-AEM-CEM-BPM BMED had a high current efficiency (90%) and low energy consumption (2.3 kW h/kg). An integrated technology of ED and BMED (Figure 9) was used by Ferrer et al. [74] to regenerate formic acid from a sodium formate and sodium hydroxide containing wastewater. ED was used to concentrate sodium formate, a three-chamber BMED was used to recover formic acid and NaOH from concentrated sodium formate, and a 30% formic acid was obtained with a current efficiency of 80% under a current density of 500 A m^−2^. Alvarez et al. [75] recovered salicylic acid (4.5 g L^−1^) in a three-chamber BMED. Wang et al. [76] used a three-chamber BMED to recover three amino acids with similar structures but different methylation groups (N-methyl glycine, N,N-dimethylglycine, and N,N,N-trimethyl glycine). The effects of feed mass concentration and current density on separation performance were analyzed in terms of molecular size, molecular structure, ion concentration, and the interaction between the amino acid and the membrane. In addition, 99.2% of itaconic acid was recovered from an itaconic acid sodium solution by Komáromy in a three-chamber BMED system (Figure 10) [77], and 92% of xylonic acid was obtained from a 100 g/L sodium xylate fermentation broth by Cao et al. [78] in a three-chamber BMED system.

**Figure 11:**
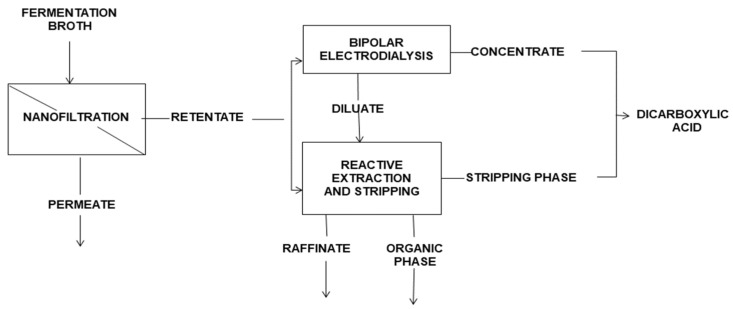
Hybrid system for the separation of fumaric acid from a fermentation broth [55].

The integration of BMED with other processes is also used to recover organic acids and bases. Prochaska et al. [55] proposed a new method (Figure 11) based on nanofiltration, BMED, and reactive extraction techniques for the recovery of fumaric acid from a fermentation broth of glycerol biotransformation, and 95% of fumarate can be concentrated and converted to fumaric acid.

## 3. CO_2_ Capture

**Figure 12:**
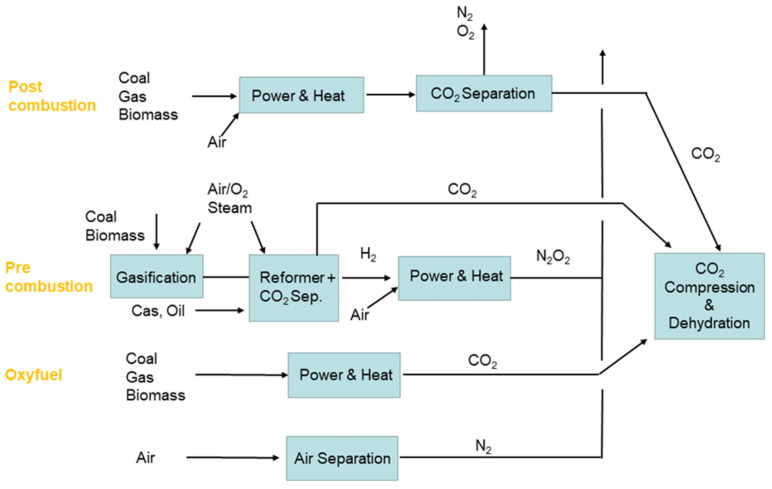
Illustration showing different approaches for capturing CO_2_ from industrial sources [79].

Global warming and carbon emissions have become a global problem, and carbon capture and storage are recognized as effective methods to reduce carbon emissions [79,80]. CO_2_ can be captured directly from industrial sources by postcombustion capture, precombustion capture, or the combustion of fossil fuels in pure oxygen environments [79] (Figure 12). Among them, postcombustion chemical absorption capture is the most mature technology [81,82,83]; however, the energy for the regeneration of capture reagents is high [84], which accounts for 70% of the cost of CO_2_ capture [85]. In the storage process, CO_2_ emitted from point sources can be captured and transported to a storage site, and the liquid CO_2_ can be injected into the ground or seawater [79]. Similarly, storage technology also involves the problems of high cost and energy consumption. It is, therefore, necessary to develop new technology to capture CO_2_.

**Figure 13:**
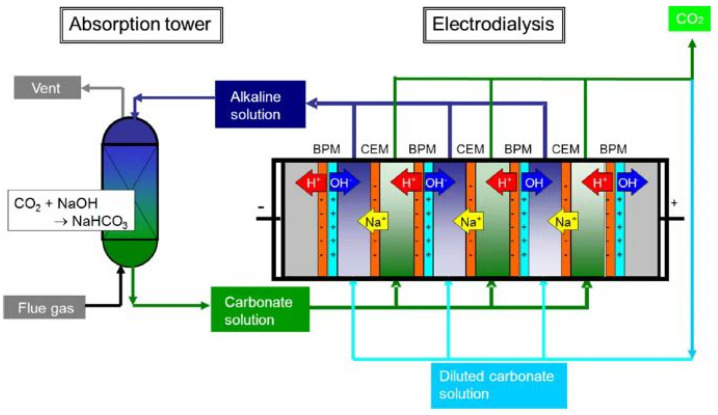
CO_2_ absorption and recovery process [87].

**Figure 14:**
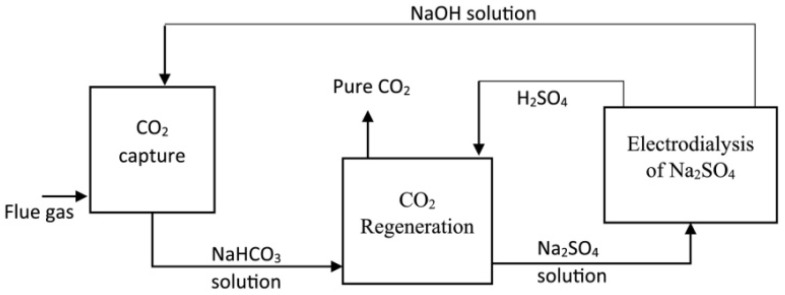
Diagram of continuous CO_2_ capture and regeneration with EDBM system. CO_2_ is captured with NaOH solution and then regenerated by reacting with H_2_SO_4_; the resultant Na_2_SO_4_ solution is subjected to electrodialysis with a bipolar membrane to produce acid and base. The base (NaOH) is circulated back to capture additional CO_2_ [84].

EDBM was demonstrated to be an effective method to capture CO_2_ from flues [86,87,88] and the atmosphere [80,89]. The main principal of CO_2_ capture by BMED is the absorption ability of bases for CO_2_, which includes the three steps shown in Figure 13 [87]. First, the CO_2_ from the flue is absorbed by NaOH to generate a carbonate solution (Equation (10)); second, the carbonate solution is sent to the BMED system, where NaOH is regenerated and gaseous CO_2_ is recovered (Equations (11) and (12)); third, the regenerated NaOH is used to capture CO_2_. However, the CO_2_ recovery is only 40–60% [87,88,90]. To increase CO_2_ recovery, Valluri et al. [84] improved the method by allowing sulfuric acid to react with sodium bicarbonate to produce sodium sulfate, and then using BMED to convert sodium sulfate to NaOH (Figure 14). In this way, 100% of CO_2_ was recovered, and energy consumption as low as 1.18 MJ/kg CO_2_ was recovered with a current efficiency of 91.2%.

NaOH _(aq)_ + CO_2 (g)_ → HCO_3_^−^ _(aq)_ + Na^+^ _(aq)_ (10)

HCO_3_^−^ _(aq)_ + H^+^ _(aq)_ → H_2_O + CO_2 (g)_↑ (11)

Na^+^ + OH^−^ → NaOH (12)

**Figure 15:**
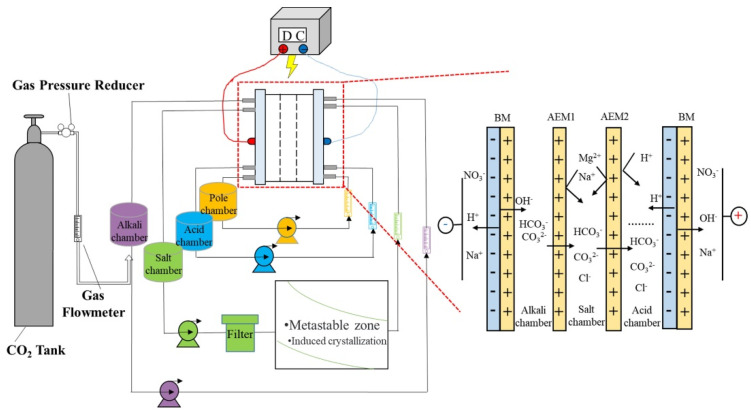
Experimental schematic diagram for magnesium extraction and CO_2_ mineralization [91].

For the capture of CO_2_ from the atmosphere, Zhao et al. [89] developed a new seawater carbon capture technology. BMED was combined with a crystallizer to avoid the precipitation of calcium ions on the membrane surface during seawater treatment. The results show that decalcification, carbon fixation, and desulfurization can reach 94.52%, 31.41% and 100%, respectively. However, the decalcified seawater still contains abundant precious magnesium resources, and the CO_2_ in the flue gas is only partially mineralized. Chen et al. [91] integrated BMED into a crystallizer to prevent the nucleation of MgCO_3_·3H_2_O and the membrane fouling problem (Figure 15), the CO_2_ fixation rate increased to 50.9%, and the magnesium recovery rate was 56.7%. Jiang et al. [80] integrated a BMED with a hollow fiber membrane (HFM) to capture CO_2_ due to HFM not only physically separating CO_2_ from liquid, but also to have a large mass transfer area [92]. The results showed that the energy consumption decreased to 2 MJ/kg CO_2_ (including heat stable salt removal). Ruan et al. [93] also integrated BMED and HFM to capture CO_2_. BMED was used to generate HCl and NaOH. HCl can be recycled to adjust the pH, and NaOH can be directly used to absorb CO_2_. Under optimal experimental conditions, the CO_2_ removal rate can reach 90.9%. Jiang et al. [94] used BMED to simultaneously capture CO_2_ and extract methionine from methionine salts. The methionine extraction rate was 99.57%, and the energy consumption for capturing 1 kg CO_2_ was 7.0 kWh.

## 4. Ammonia Nitrogen Production and Recovery

With the rapid development of the economy and industrialization, industrial wastewater containing ammonia is continuously discharged into the environment, causing serious environmental problems [95]. According to the study by Erisman et al. [96], half of the NH_3_ produced enters into the environment, leading to eutrophication and a subsequent loss of biodiversity. Therefore, ammonia pollution has become a serious environmental problem in many industrialized countries, and it is urgent to develop effective ammonia removal technologies. Nanofiltration, reverse osmosis, and biodegradation technologies have been studied to treat ammonia wastewater [97,98,99], but these methods involve high energy consumption, secondary pollution, and high costs.

**Figure 16:**
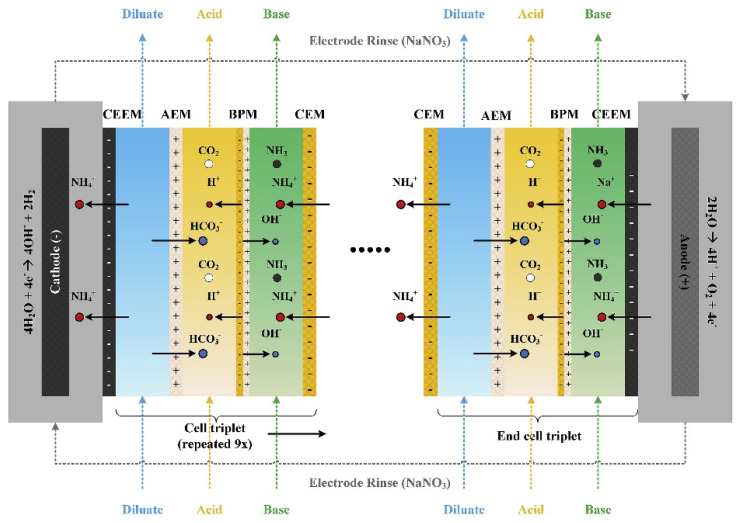
The membrane and (flow) cell sequence in the BMED and the intended ion transport (electromigration and water dissociation) as a result of the applied current. In the acid, H^+^ and HCO_3_^−^ are combined and react to CO_2_, while in the base, OH^−^ and NH_4_^+^ are combined and react to NH_3_. At the cathode, NH_4_^+^ is transported to the electrode rinse, while at the anode, both Na^+^ and NH_4_^+^ are transported to the base, resulting in the accumulation of NH_4_^+^ in the electrode rinse and the washing out of Na^+^ to the base [100].

In recent years, BMED has often been used to recover ammonia from wastewater. Linden et al. [100] recovered HCl and NH_3_·H_2_O from simulated NH_4_Cl wastewater by BMED (Figure 16). The effects of initial NH_4_Cl concentration, current density, salt solution volume, initial acid–base concentration, and membrane stack structure on the yields of HCl and NH_3_·H_2_O were investigated. The total ammonia removal efficiency was between 85% and 91%, and the energy consumption was stable at 19 MJ kg^−1^ of nitrogen, which is more competitive than ED combined with added chemicals (22 MJ kg N^−1^ of nitrogen). The effects of humic acid, Mg^2+^, and Ca^2+^ on the removal of ammonium were investigated by Zhang et al. [101], with the results showing that humic acid and a high concentration of Mg^2+^ and Ca^2+^ has a significant effect on ammonium removal: more than 86% of ammonium was removed from a leachate collected from a local landfill. In addition, Ferrari et al. [102] combined an on-site BMED with two liquid/liquid membrane contactors to recover ammonia from an anaerobic fermentation broth. Graillon et al. [103] found that a BMED system is not suitable for recovering nitric acid and ammonia from an ammonium nitrate solution; they proposed a two-step process instead. First, NaOH is added to the ammonium nitrate solution and ammonia is stripped, then the obtained sodium nitrate solution is treated by BMED to regenerate nitric acid and NaOH in a second step. However, this increases the operational difficulty and cost. Ali et al. [104] achieved 83% recovery of ammonium from an ammonium nitrate solution using an integrated technology of BMED and continuous in situ ammonia stripping. The different results may be caused by the differences in the BMED configuration and operation process.

From the above, we note that ammonia can be recovered from ammonium-containing wastewater, but the phenomena of OH^−^ leakage, dissolved ammonia diffusion, and ionic diffusion from the base to the diluent occur during the BMED process, which can decrease the current efficiency. With the extension of the experimental time and the increase in concentration gradients between the diluent and concentrated compartments, the current efficiency decreased from 69% to 54% [100]. This is the deficiency of BMED technology in the treatment of ammonium-containing wastewater.

## 5. Ion Removal and Recovery from Wastewater

**Figure 17:**
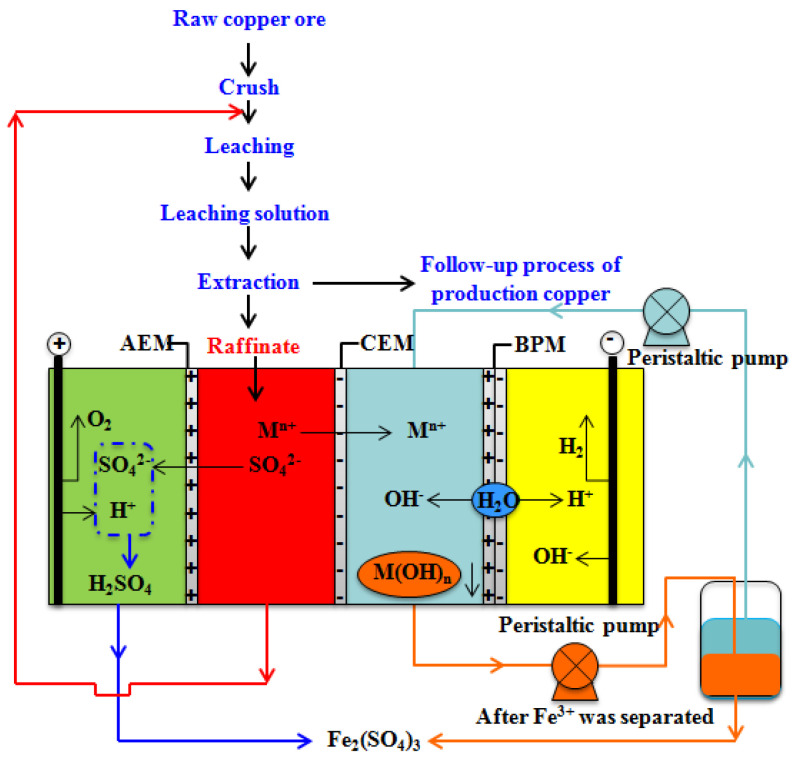
Schematic representation of the BMED experimental setup for treating raffinate. AC, anode chamber; RC, raffinate chamber; HMC, heavy metal chamber; CC, cathode chamber; AEM, anion-exchange membrane; CEM, cation-exchange membrane; BPM, bipolar membrane [107].

With the improvement of environmental standards and the shortage of resources, the removal and recovery of metals and valuable ions from wastewater have both environmental protection and economic benefits. Heavy metals are a class of harmful pollutants, which are toxic, carcinogenic, nonbiodegradable, and persistent in the environment and organisms [105]. Once they enter the food chain, they accumulate in the human body and lead to serious health problems [104]. The most common method for removing heavy metals from wastewater is chemical precipitation, but a large amount of sludge can be generated that is hard to treat [105]. Wu et al. [106] used an improved BMED system combined with H_2_O_2_ oxidation to recover Cr(III) from a sodium chromate solution, and 87.8% of Cr(III) was recovered. Raffinate produced during the hydrometallurgical processing of copper ore has strong acidity and a high content of heavy metals (iron, zinc, copper, etc.), and so is difficult to treat. Liu et al. [107] found that 99.3% of iron, 99.1% of zinc, 99.0% of copper, 84.9% of nickel, 70.6% of chromium, 95.8% of cadmium, and 94.8% of arsenic can be removed, and 85.9% of SO_4_^2−^ can be recovered in the form of H_2_SO_4_, in a BMED system (Figure 17). Liu et al. [108] also used a BMED system to remove arsenic and cationic metals from copper slag produced during copper ore hydrometallurgy, with only a small amount of stable residual metals remaining in copper slag after treatment.

**Figure 18:**
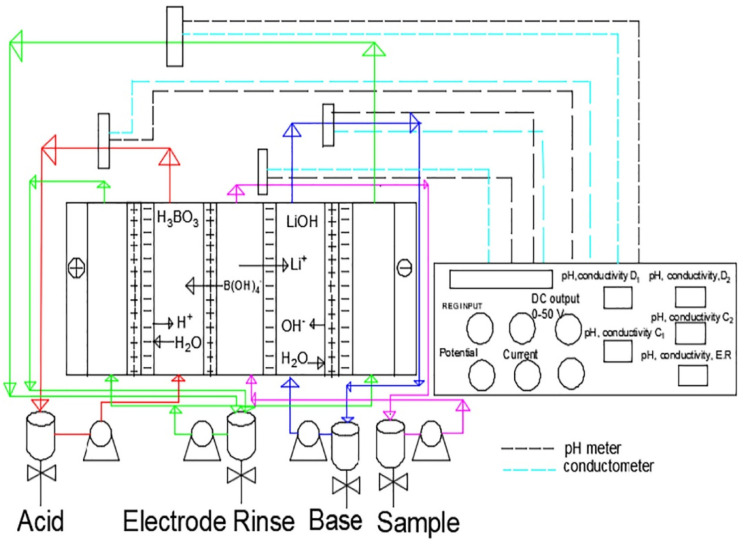
The flow scheme of a BMED system [111].

In recent years, BMED has often been used to recover boron and lithium from solutions in the form of H_3_BO_3_ and LiOH. Sun et al. [109] introduced a QGO-P84 membrane into BMED to recover boron from a synthetic model solution (Na_2_B_4_O_7`_10H_2_O, 1000 mg B/L); the recovery rate of boron and the energy consumption were 94.9% and 26.16 kW h/kg, respectively. In the study by Jarma et al. [110], 38.8% of boron and 50.2% of lithium were recovered in a BMED system. İpekçi et al. [111] also used a BMED system to separate and recover boron and lithium from aqueous solutions (Figure 18). The results showed that the separation efficiency and recovery of boron and lithium increased when the potential increased from 15 V to 25 V under a solution flow rate of 50 L/h, and the removal and recovery rates were 74.4% and 59%, while those of lithium were 99.0% and 73.0%, respectively. İpekçi et al. [112] separated and recovered lithium and boron from an aqueous solutions using a BMED. The separation rates of boron and lithium were 86.9% and 94.7%, respectively, with a specific energy consumption of 7.9 kWh/m^3^. In the study by Bunani et al. [113], the separation and recovery of lithium were 99.6% and 88.3%, respectively, while those of boron were 72.3% and 70.8%, respectively. Bunani et al. [114] recovered 39.1% of boron and 20.0% of lithium using a BMED system. In the study by Hung et al. [115], the removal and recovery rates of boron were 98.6% and 86.5%, respectively, in a BMED system.

From the above, we conclude that BMED can be used to remove and recover heavy metals from wastewater. Additionally, the recovery of boron and lithium from solutions using a BMED system has good application prospects. It was also found that the removal and recovery of boron and lithium are different in different studies, mainly because of difference in BMED size, membrane performance, wastewater properties, and operating conditions. The studies on boron and lithium removal and recovery are summarized in Table 2 and Table 3, respectively.

## 6. Conclusions

At present, BMED technology has been widely used for recovering NaCl, NaSO_4_, and NaNO_3_ salts in the form of acids and bases. In terms of organic acids, after more than 10 years of research and development, it has moved toward large-scale production. CO_2_ capture, ammonia nitrogen recovery, wastewater ion removal and recovery are still at the lab scale; however, with increased attention being paid to these fields, it can be speculated that BMED technology will have good application.

In addition, BMED has some problems that limit its development, such as a high preparation cost, and membrane fouling and ion leakage during operation. The cost and performance of BMED depend on the membrane characteristics, feed conditions, required product quality, and parameter design such as stack structure and current density. The problem of membrane fouling during operation can be solved through modifications to the membrane hydrophilicity, charge, and roughness by adding modified components (such as nanoparticles), by pretreating the raw material solution (such as using chemical precipitation, coagulation, filtration, and other methods to reduce the solution ion concentration), and by changing the operating conditions (such as the feed solution pH, raw material solution concentration, feed speed, and other factors). To solve the problem of ion leakage, an acid-blocking membrane can be used to block the migration of H^+^. H^+^ migration can also be reduced by controlling the cell voltage, current density, and salt compartment solution pH. Alternatively, AEM can be added to the three-chamber BMED to reduce the competitive migration of H^+^ with other cations. Although BMED has some deficiencies, its characteristics, such as environmental friendliness, no need to add chemical reagents, small footprint, high efficiency, and simple operation, mean it has been widely used in the field of environmental protection and resources recovery. It can be predicted that with the gradual deepening of the research on BMED, it will be applied in more fields.

## Figures and Tables

**Table 1:** A comparison of BMED and other membrane processes.

Type	ED	Ion Exchange	BMED
additive	consume a lot of acid	consume a lot of acid and base	does not consume any acid and base
product	produce a lot of salt waste liquid, the produced high-soda ash solution can be recycled	produces a lot of acid and base waste liquid, and it is difficult to recover	no waste liquid is produced, and the produced high-soda ash solution can be recycled
production scale	capable of mass production	not suitable for mass production	capable of mass production of inorganic acids and bases; the market for organic acids and bases is expanding
recycling effect	high recovery and high purity	the recovery efficiency is low; the purity is high	high recovery efficiency and purity

**Table 2:** Separation and recovery of boron from aqueous solutions using BMED.

Method	Operating Conditions	Boron Concentration	Efficiency	References
intermittent BMED	voltage: 12 V, current density: 6.36 A/m^2^, pH: 9.5–10.5, solution flow rate: 36 L/h	680 mg B/L	S_B_ = 98.6% β_B_ = 86.5%	[115]
Continuous BMED	Voltage: 12 V, current density: 6.36 A/m^2^, pH: 9.5–10.5, solution flow rate: 36 L/h reaction time: 5 cycle	612 mg B/L	S_B_ = 98.53% β_B_ = 81.2%	[115]
BMED	voltage: 30 V, reaction time: 3 h, membrane: QGO-P84 membrane	1000 mg B/L	S_B_ = 76.6%	[109]
	voltage: 30 V, reaction time: 3 h, membrane: Commercial membrane CJMA-3	1000 mg B/L	S_B_ = 51.6%	[109]
	voltage: 15 V, sample solution: 0.5 L 3 mM HCl-3 mM NaOH membrane: PC-bip membranes	850 mg B/L	S_B_ = 72.3% β_B_ = 70.8%	[113]
	voltage: 30 V, type of membrane: AHA membranes	1000 mg B/L	S_B_ = 97.8% β_B_ = 39.1%	[114]
	voltage: 30 V, sample solution: 2 L 5 mM HCl-5 mM NaOH	1000 mg B/L	S_B_ = 86.9% β_B_ = 50.0%	[112]
	voltage: 20 V, sample solution: 0.05 mol/L H_3_BO_3_-0.05 mol/L LiOH solution flow rate: 45–50 L/h, membrane: PC-Cell ED 640 04 model	924 mg B/L 313 mg Li/L	S_B_ = 77.5% β_B_ = 54.0%	[110]
	voltage: 20 V, sample solution: 0.05 moL/L H_3_BO_3_, 0.05 mol/L LiOH solution flow rate: 45–50 L/h, membrane: Mega EDR-Z-Full-V4 model	976 mg B/L, 314 mg Li/L	S_B_ = 81.0% β_B_ = 38.8%	[110]
	voltage: 25 V, sample solution: 3 mM HCl-3 mM NaOH solution flow rate: 50 L/h, membrane: Mega EDR-Z-FULL-V4 model	812 ± 56.15 mg B/L	S_B_ = 74.0% β_B_ = 59.0%	[111]

S_B_: Separation rate of boron; β_B_: Recovery rate of boron.

**Table 3:** Separation and recovery of lithium from aqueous solutions using BMED.

Method	Operating Conditions	Feed Solution	Efficiency	References
BMED	Voltage: 15 V, sample solution: 0.5 L 3 mM HCl, 3 mM NaOH membrane: PC-bip membranes	250 mg Li/L	S_Li_ = 99.6% β_Li_ = 88.3%	[113]
	Voltage: 30 V, sample solution: 0.1 M HCl, 0.1 M NaOH membrane: AHA BP-1E membranes	340 mg Li/L	S_Li_ = 97.8% β_LI_ = 20.0%	[114]
	Voltage: 30 V, sample solution: 2 L 5 mM HCl, 5 mM NaOH	340 mg Li/L	S_Li_ = 94.7% β_Li_ = 62.0%	[112]
	Voltage: 20 V, sample solution: 0.05 moL/L H_3_BO_3_, 0.05 moL/L LiOH solution flow rate: 45–50 L/h, membrane: PC-Cell ED 640 04 model	313 mg Li/L	S_Li_ = 99.8% β_Li_ = 86.4%	[110]
	Voltage: 20 V, sample solution: 0.05 mol/L H_3_BO_3_, 0.05 mol/L LiOH solution flow rate: 45–50 L/h, membrane: Mega EDR-Z-Full-V4 model	314 mg Li/L	S_Li_ = 99.8% β_Li_ = 50.2%	[110]
	Voltage: 25 V, sample solution: 3 mM HCl, 3 mM NaOH solution flow rate: 50 L/h, membrane: Mega EDR-Z-FULL-V4 model	256 ± 33.11 mg Li/L	S_Li_ = 99.0% β_Li_ = 73.0%	[111]

S_Li_: Separation rate of lithium; β_Li_: Recovery rate of lithium.

## Data Availability

Not applicable.
